# Herbal Formula Extract Ameliorates Anxiety and Cognitive Impairment via Regulation of the Reelin/Dab-1 Pathway in a Murine Model of Post-Traumatic Stress Disorder

**DOI:** 10.3390/pharmaceutics16091150

**Published:** 2024-08-30

**Authors:** Hee Ra Park, Mudan Cai, Eun Jin Yang

**Affiliations:** Department of KM Science Research, Korea Institute of Oriental Medicine (KIOM), Daejeon 34054, Republic of Korea; hrpark0109@kiom.re.kr (H.R.P.); mudan126@kiom.re.kr (M.C.)

**Keywords:** post-traumatic stress disorder, herbal medicine, hippocampus, Reelin, DNA methylation

## Abstract

We investigated the effects of epigenetic modifications on post-traumatic stress disorder (PTSD) using a novel combination of herbal medicines from *Panax ginseng*, *Astragalus membranaceus*, *Atractylodes macrocephala*, and *Glycyrrhiza uralensis*. The herbal formula extract (HFE) (250 mg/kg) was administered orally once daily for 14 days to determine its effects on PTSD in mice by combining prolonged stress and foot shock. The open field and Y-maze tests determined the effect of HFE on PTSD-induced anxiety and cognition. Hippocampal neuronal plastic changes and molecular mechanism were verified. Treatment with HFE decreased anxiety-like behavior and enhanced cognition. Moreover, it reduced the number of PTSD-related hilar ectopic granule cells in the dentate gyrus (DG). PTSD mice showed reduced neuronal plasticity of doublecortin^+^ cells in the DG, which was restored by HFE treatment. HFE reversed PTSD-induced inhibition of the Reelin/Dab1 pathway, a critical signaling cascade involved in brain development, and regulated *Reelin* methylation. Furthermore, DNA methylation, methyl-CpG binding protein 2, and DNA methyltransferase 1, which were elevated in the hippocampus of PTSD mice, were restored following HFE treatment. HFE increased the expression of synaptic plasticity-related factors in the hippocampus of PTSD mice. Our findings suggest that HFE can facilitate PTSD treatment by alleviating behavioral abnormalities through the restoration of hippocampal dysfunction via regulation of the Reelin/Dab-1 pathway and DNA methylation in the hippocampus.

## 1. Introduction

Post-traumatic stress disorder (PTSD) is a complex neuropsychiatric disorder associated with traumatic memories and anxiety [1]. People with PTSD experience intrusive memories, depressed moods, flashbacks, nightmares, and avoidance behaviors. The hallmark symptoms of PTSD involve alterations to cognitive processes such as learning, working memory, and attention, which are associated with dysregulation in the dentate gyrus (DG) of the hippocampus [2]. Structural magnetic resonance imaging (MRI) studies in patients with PTSD have reported that reduction in DG volume is correlated with PTSD symptoms [3]. In addition, PTSD animal models have shown structural and functional changes in hippocampal neurons, such as abnormal neuronal plasticity and aberrant neuronal location [4,5]. However, despite the association between PTSD and dysregulated changes in the DG, the cellular mechanisms of the DG that are responsible for cognitive impairment in PTSD remain unclear.

Epigenetic regulation, such as DNA methylation, has been known to be related to hippocampal function and cognitive processes [6,7]. Higher DNA methylation levels have been reported in the blood samples of patients with PTSD compared with those of healthy individuals, which can affect cellular mechanisms by regulating gene expression, and this has been reported to be closely related to PTSD symptoms such as anxiety, fear, and cognitive impairment [8,9]. These findings suggest the potential for exploring therapeutic drugs for PTSD through the regulation of DNA methylation [10].

Reelin is an extracellular glycoprotein regulated in key cellular pathways in the developing and adult brain, including neuronal migration, neuronal positioning, synapse formation, and synaptic plasticity [11]. These roles are mediated by the intracellular phosphorylation of disabled-1 (Dab-1) downstream of the Reelin pathway [11]. Mice with a spontaneous Reelin mutation demonstrate symptoms such as ataxia, hypotonia, and ectopic neuronal migration, resulting in disrupted dendritic trees and axonal projection, especially in the cortex and hippocampus [12,13,14]. Interestingly, genetic deletion of Reelin resulted in mice that exhibited neuropsychiatric disorders, cognitive impairment, and social-seeking behavior deficits [15,16]. Furthermore, various stressors, including social isolation, repeated corticosterone administration, and chronic unpredictable mild stress, led to a decrease in Reelin expression in the hippocampus, which was accompanied by cognitive dysfunction and mental health problems [17,18]. Treatment with recombinant Reelin protein in the brain exhibited therapeutic effects on memory deficit, loss of immobility, and long-term potentiation (LTP) impairment in rats with corticosterone-induced stress or mice with MK-801-induced memory impairment [17,19]. The Reelin/Dab-1 pathway dynamically induces gene transcriptional changes in neurons, and their target genes are positively correlated with genes implicated in neuropsychiatric disorders and hippocampal function [20]. However, the role of Reelin in PTSD remains unclear.

The diverse biological efficacies of herbal medicines can help alleviate symptoms of PTSD through several mechanisms [21]. One of these is the regulation of DNA methylation, which affects the expression of epigenetic modifiers such as methyl-CpG-binding protein 2 (MeCP2) and DNA methyltransferase 1 (DNMT1) [22]. We designed a novel herbal formula extract (HFE), which included *Panax ginseng*, *Astragalus membranaceus*, *Atractylodes macrocephala*, and *Glycyrrhiza uralensis*, all recognized for their neuroprotective, anti-inflammatory, antioxidant, and anticancer activities, to address whether treatment with herbal medicine rescues hippocampal dysfunction and abnormal behaviors in PTSD mice [23,24,25,26]. The aim of this study was to demonstrate the effects of this novel HFE on anxiety, cognitive impairment, hippocampal dysfunction, and regulation of reelin methylation in the hippocampus of PTSD mice.

## 2. Materials and Methods

### 2.1. Establishment of the PTSD Mouse Model

**Figure 1:**
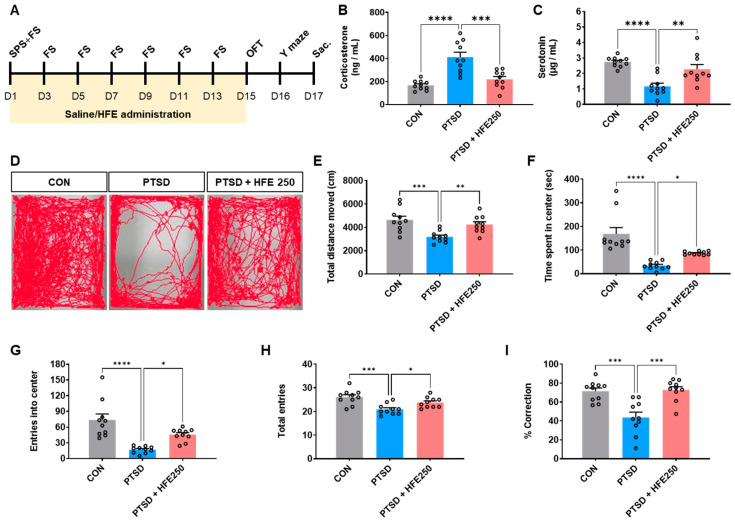
HFE administration ameliorates PTSD-like behavior in PTSD mice. Mice were subjected to behavioral tests after HFE administration for 14 days. (**A**) In vivo experimental scheme. (**B**) Quantitative analysis of the corticosterone concentration in the serum of each group. (**C**) Quantitative analysis of the serotonin concentration in the serum of each group. (**D**) Movement traces of mice in the open field test. (**E**) Total distance moved in the open field test. (**F**) Time spent in the central zone of the open field test. (**G**) Entries in the central zone of the open field test. (**H**) Total number of entries in the three arms of the Y-maze test. (**I**) The percentage of spontaneous alternation in three arms in the Y-maze test. Data are expressed as the mean ± SEM (*n* = 10 mice/group); * *p* < 0.05, ** *p* < 0.01, *** *p* < 0.001, **** *p* < 0.0001. CON, control mice; FS, foot shock; HFE, herbal formula extract; OFT, open field test; PTSD, post-traumatic stress disorder; SPS, single prolonged stress; SEM, standard error of the mean.

Six-week-old C57BL/6J male mice were purchased from Daehan BioLink (Eumseong-gun, Chungcheongbuk-do, Republic of Korea). All mice were housed under temperature- and light-controlled conditions (20−23 °C, 12/12 h light/dark cycle), with access to food and water ad libitum, and were acclimatized for 7 days. They were housed in groups of five mice per cage. Ethical approval for the in vivo study was obtained from the Institutional Animal Care and Use Committee (IACUC) of the Korea Institute of Oriental Medicine (KIOM) (Approval No.: #22-108) and followed the KIOM animal care guidelines. The PTSD mouse model was adapted from the single prolonged stress (SPS) protocol [27], involving a combination of stressors: 4 h of physical restraint followed by 20 min of forced swimming. Following a 15-min recovery period, mice were exposed to diethyl ether till unconsciousness (≤ 1 min). Subsequently, the animals received electric foot shocks (1 mA for 5 s, twice every 2 days) using a shock chamber (Startle and Fear combined system, Harvard Apparatus, Holliston, MA, USA). The in vivo experimental design is illustrated in Figure 1A.

### 2.2. Preparation of HFEs

Medicinal herbs, including *P. ginseng*, *A. membranaceus*, *A. macrocephala*, and *G. uralensis*, were sourced from Kwangmyungdang Medicinal Herbs Co. (Ulsan, Republic of Korea). Water extracts of these herbs, mixed at a 1:1:1 ratio, were prepared by KOC BIOTECH (Daejeon, Republic of Korea). Mixed herbs (50 g) were extracted with 1000 mL of distilled water in a shaking incubator for 24 h at 40 °C, followed by filtration and concentration under vacuum. The resulting water extracts were subsequently freeze-dried to obtain a powdered extract, which was freshly dissolved in 0.9% physiological saline before administration.

### 2.3. HFE Administration

A total of 30 mice were divided randomly into three groups as follows: (1) Control (CON, unstressed mice, *n* = 10), (2) PTSD (SPS+FS-induced stressed mice, *n* = 10), and (3) PTSD+HFE 250 mg/kg (*n* = 10). Freshly dissolved HFE in 0.9% physiological saline was administered orally for 14 consecutive days. Control and PTSD groups received an equivalent oral volume of 0.9% physiological saline.

### 2.4. Open Field Test (OFT)

The OFT was conducted according to established methods [28]. Mice were placed individually in the OFT arena and allowed to freely navigate the arena for 20 min. EthoVision XT video tracking software (Version XT 8.5, Noldus, Wageningen, The Netherlands) was used to conduct and analyze the OFT. The OFT box was cleaned with 70% ethanol and dried after each trial.

### 2.5. Y-Maze Test

The Y-maze test was conducted following previously described procedures [28]. Each mouse was given 8 min to freely explore the Y-maze. The number of spontaneous alternations was defined as sequential entry into three different arms (ABC, BCA, or CAB). The percentage of alternation was calculated as [number of spontaneous alternations/(total number of arm entries − 2)] × 100 (%). The Y-maze was cleaned with 70% ethanol and dried after each trial.

### 2.6. Tissue Preparation

On day 17, mice were anesthetized using avertin (2,2,2-tribromoethanol, Sigma-Aldrich, St. Louis, MO, USA). Serum for corticosterone and serotonin assays was obtained by centrifuging blood at 3000 rpm for 15 min at 4 °C. For Western blot analysis, hippocampi were homogenized in radioimmunoprecipitation assay (RIPA) buffer (Millipore, Burlington, MA, USA) supplemented with Xpert Duo Inhibitor Cocktail Solution (GenDEPOT, Katy, TX, USA) and centrifuged at 12,000 rpm for 20 min at 4 °C. The serum and hippocampi lysates were stored at −80 °C until further use. For histological analyses, mice were perfused intracardially with 4% PFA in 0.1 M phosphate-buffered saline (PBS; pH 7.4). Brains were post-fixed at 4 °C overnight and transferred to a 30% sucrose solution. Coronal cryosections of 40 μm thickness were obtained using a freezing microtome (Leica Biosystems, Wetzlar, Germany). All sections were stored in Dulbecco’s PBS (DPBS) containing 0.1% sodium azide at 4 °C.

### 2.7. Serum Corticosterone and Serotonin Assays

Levels of corticosterone and serotonin were assessed using specific assay kits: the corticosterone assay kit (#EIACORT, Thermo Scientific, Waltham, MA, USA) and the serotonin assay kit (#MBS1601042, Mybiosource, San Diego, CA, USA), following the manufacturer’s instructions. The absorbance at 450 nm was taken using a microplate reader (SpectraMax i3; Molecular Devices, CA, USA), and concentrations were determined based on standard curves.

### 2.8. Immunostaining

Immunostaining procedures were conducted as described previously [28]. Sections were stained with specific primary antibodies. Images were captured using an optical microscope (BX53, Olympus, Tokyo, Japan), and confocal z-stack images were acquired with an FV10i FLUOVIEW confocal scanning microscope (Olympus). The specific primary antibodies used in this study include proliferating cell nuclear antigen (PCNA, mouse, #2586; Cell Signaling Technology, Danvers, MA, USA), doublecortin (DCX, 1:500, rabbit, #40619; Cell Signaling Technology), Prox1 (1:500, rabbit, #ab199359, Abcam, Cambridge, MA, USA), NeuN (1:500, mouse, #MAB377, Sigma-Aldrich), Reelin (1:500, mouse, #MAB5364, Sigma-Aldrich), and phosphorylated (p)-CREB (Ser 133, 1:500, rabbit, Abcam) antibodies. A quantitative analysis of histological data was performed by investigators blinded to all image results.

### 2.9. Western Blot Analysis

Western blotting was conducted following established protocols [28]. Proteins were detected using an enhanced chemiluminescent (ECL) substrate (SuperSignal^TM^ West Femto, Thermo Scientific) and imaged with a ChemiDoc Touch Imaging System (Bio-Rad Laboratories, Hercules, CA, USA). Band intensities were quantified using Image Lab software (version 6.1.0, Bio-Rad). The specific primary antibodies used for Western blot analysis included DCX (1:1000, rabbit, #40619, Cell signaling Technology), Prox1 (1:1000, rabbit, #ab199359, Abcam), Reelin (1:1000, mouse, #sc-25346, Santa Cruz Biotechnology, CA, USA), p-Dab1 (Tyr 220, 1:1000, rabbit, #BS-3114R, Bioss Antibodies, Woburn, MA, USA), p-Dab1 (Tyr 232, 1:1000, rabbit, #BS-3115R, Bioss Antibodies), total(t)-Dab1 (1:1000, mouse, #sc-271136, Santa Cruz Biotechnology), p-Akt (Ser 473, 1:1000, rabbit, #9271, Cell signaling Technology), t-Akt (1:1000, rabbit, #9272, Cell signaling Technology), p-GSK3β (Ser 9, rabbit, #MA5-14873, Invitrogen, Waltham, MA, USA), t-GSK3β (1:1000, mouse, #39-9500, Invitrogen), p-mTOR (Ser 2448, rabbit, #5536, Cell signaling Technology), t-mTOR (1:1000, rabbit, #2983, Cell signaling Technology), p-ERK (Thr202/Tyr204, 1:1000, rabbit, #4370, Cell Signaling Technology), total (t)-ERK (1:1000, rabbit, #9102, Cell Signaling Technology), BDNF (1:1000, rabbit, #PA5-85730, Invitrogen), and α-tubulin (1:5000, mouse, #MA5-31466, Invitrogen).

### 2.10. Reverse Transcription-Quantitative Polymerase Chain Reaction (RT-qPCR)

Total RNA was isolated from each hippocampus tissue using a total RNA extraction kit (easy-spin^TM^ Total RNA Extraction Kit, #17221, iNtRON Biotechnology, Seongnam-si, Republic of Korea) as described by the manufacturer. The concentration of total RNA was measured using NanoDrop One (Thermo Scientific). According to the manufacturer’s protocol, cDNA was synthesized using a cDNA synthesis kit (iScriptTM cDNA Synthesis Kit, #1708891, Bio-Rad). Each cDNA template was combined with SYBR Supermix (iTaq^TM^ Universal SYBR^®^ Green Supermix, #1725121, Bio-Rad), gene-specific primers, and nuclease-free water. qPCR was performed using a QuantStudio^TM^ 6 Flex (Life Technologies, Carlsbad, CA, USA) with the following cycle conditions: 95 °C for 30 s, followed by 40 cycles of denaturation at 95 °C for 15 s, and 60 °C for 1 min. Glyceraldehyde-3-phosphate dehydrogenase (*GAPDH*) was used as the housekeeping gene for normalization. Gene-specific primers used in the qPCR analysis were synthesized by Bioneer (Daejeon, Republic of Korea) and are listed in Table 1. These primers were designed to amplify specific target genes relevant to the study.

### 2.11. Bisulfite Modification and PCR of Genomic DNA

This assay is based on converting non-methylated cytosine to uracil through bisulfite modification, which leaves the methylated cytosine unaltered. The protocol was performed according to da Silva et al. [29]. Briefly, genomic DNA (gDNA) was isolated from the hippocampus of each mouse using genomic DNA kits (PureLink^®^ Genomic DNA Kits, #1820-02, Invitrogen), and 350 ng of gDNA was bisulfite-modified using a bisulfite conversion kit (EpiJET Bisulfite Conversion Kit, #K1461, Thermo Scientific) following the manufacturer’s instructions. PCR reactions were carried out in a final volume of 25 μL, containing methylation-specific PCR premix (AccuPower^®^ Epigene^TM^ methylation-specific PCR premix, #K2410, Bioneer), 80 ng bisulfite modified gDNA from each mouse, and 10 pmol each of the unmethylated or methylated primers. The primer sequences are shown in Table 1. PCR cycling conditions included a pre-denaturation step at 95 °C for 5 min, followed by 40 cycles of denaturation at 95 °C for 30 s, 56 °C for 30 s, and 72 °C for 1 min; this finished with a final extension step at 72 °C for 5 min. PCR products (2 μL each) were analyzed by electrophoresis on a 0.8% agarose gel stained with RedSafe^TM^ nucleic acid staining solution (#21141, iNtRON Biotechnology) and directly visualized under UV light illumination using ChemiDoc Touch Imaging System (Bio-Rad). The relative band intensities were calculated using Image Lab software version 6.1.0 (Bio-Rad). The percentage of methylation index was calculated as [(intensity of methylation/intensity of methylation + intensity of unmethylation)] × 100 (%).

### 2.12. Quantitative DNA Methylation Assay

Global DNA methylation was measured using a methylated DNA quantification kit (#ab117128, Abcam) according to the manufacturer’s instructions. Initially, 80 μL of the binding solution was added to the provided strip wells. Subsequently, 1 μL of negative control, 1 μL of positive control, and 100 ng of gDNA were added in duplicate to the wells. The plate was incubated for 90 min at 37 °C with shaking. After the plate was washed, 150 μL of capture antibody solution was added to each well and incubated for 1 h at room temperature. After the plate was washed, 50 μL of detection antibody solution was added to each well and incubated for 30 min at room temperature. After the plate was washed, 50 μL of enhancer solution was added to each well and incubated for 30 min at room temperature. After the plate was washed and dried, 100 μL of the developer solution was added to each well and incubated at room temperature for 1–10 min away from light. The reaction was terminated by adding 50 μL of the stop solution, and the optical density (OD) was read at a 450 nm wavelength using a microplate reader. The absolute amount of methylated DNA was determined using the formulae provided by the manufacturer.

### 2.13. High Performance Liquid Chromatography (HPLC)/Photodiode Array Detector (PDA) Analysis of HFE

HFE (40 mg) was completely dissolved in 2 mL of 100% methanol. After filtration through a 0.45 μm polyvinylidene fluoride microfilter (Whatman, Maidstone, UK), the active compounds of HFE were analyzed using the Waters e2695 HPLC System (Waters Corporation, Milford, MA, USA) coupled with an ultraviolet (UV) detector set at 220 nm and a 2998 photodiode array detector (PDA, 200−450 nm). An Inno C18 column (4.6 × 250 mm; particle size, 5 μm; Young Jin Biochrom Co., Ltd., Gyeonggi-do, Republic of Korea) was used. The mobile phase consisted of 0.1% (*v*/*v*) trifluoroacetic aqueous solution (A) and acetonitrile (B). The elution conditions for identifying HFE, 2-(4-hydroxyphenyl)ethanol, methyl 3,4,5-trihydroxybenzoate, liquiritin, fisetin, and glycyrrhizic acid were as follows: starting at 0 min with 90% A/10% B held for 10 min, followed by a gradient to 40% A/60% B over 11–60 min, then a 10 min wash with 100% B, and finally a 10 min equilibration period at 90% A/10% B. The separation temperature was maintained at 40 °C throughout the analysis, with a flow rate of 1.0 mL/min and an injection volume of 20 μL. Compound identification was based on retention time and UV spectra compared with commercial standards.

### 2.14. Statistical Analyses

Data were analyzed using one-way ANOVA followed by Dunnett’s test using GraphPad PRISM software^®^ (GraphPad PRISM software Inc., Version 9.4.1, San Diego, CA, USA). Results are presented as the mean ± standard error of the mean (SEM). Statistical significance was defined as *p* < 0.05.

## 3. Results

### 3.1. Effect of HFE Administration on Serum Corticosterone and Serotonin Levels in PTSD Mice

We established a mouse model by exposing mice to mixed stress with SPS+FS and administered HFE (250 mg/kg) for 14 days and analyzed the corticosterone and serotonin levels in the serum to verify the usefulness of the model. The corticosterone concentration in the serum was higher in PTSD mice than in CON mice. However, HFE treatment suppressed serum corticosterone (Figure 1B). The serotonin concentration in the serum was lower in PTSD mice than in CON mice. In contrast, HFE treatment increased the serotonin concentration in the serum (Figure 1C).

### 3.2. HFE Administration Ameliorates PTSD-like Behavioral Abnormalities

We performed OFT and Y-maze tests to investigate whether HFE can alleviate SPS+FS-induced behavioral abnormalities, such as anxiety, cognitive deficit, and fear response, after 14 days of HFE treatment. In the OFT, mice in the PTSD group showed reduced locomotor activity by spending less time and entering less frequently into the central zone than did the CON mice, indicating that stressed mice exhibit anxiety-like behavior (Figure 1D−G). Treatment with 250 mg/kg HFE significantly recovered the total moving distance in the OFT. Further, the time spent and the number of entries in the central zone were increased in the 250 mg/kg HFE-treated group in the OFT. The Y-maze test showed a decrease in the total number of entries into all three arms in PTSD mice compared with CON mice, consistent with the decrease in locomotor activity observed in OFT (Figure 1H). However, HFE administration increased the number of total entries into the arms in the Y-maze test. Compared with CON mice, PTSD mice showed a significantly reduced percentage of spontaneous alternation by revisiting the recently visited arm, but they recovered after HFE treatment (Figure 1I).

### 3.3. Hippocampal Dysfunctions in PTSD Mice Are Reduced by HFE Administration

**Figure 2:**
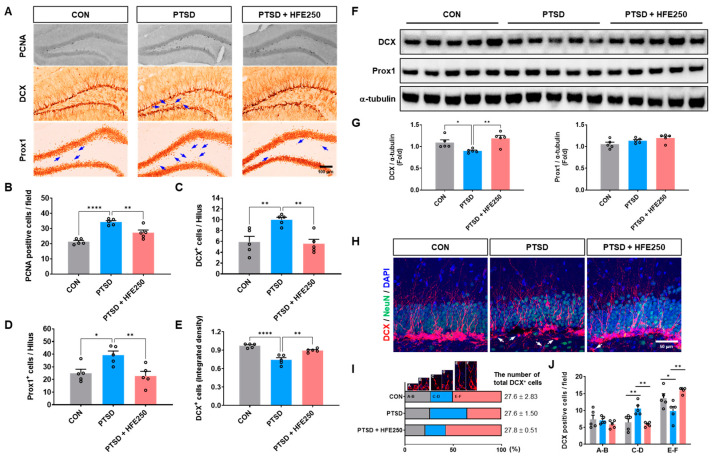
Administration of HFE reduces hilar ectopic DGCs and increases dendritic morphologies in the DG of PTSD mice. (**A**) Representative images of PCNA, DCX, and Prox1 in the DG. Arrows indicate DCX^+^ and Prox1^+^ cells in the hilus. Scale bar: 100 μm. (**B**) Quantification of PCNA^+^ cells in the SGZ of the DG of each group (*n* = 10 sections/5 mice/group). (**C**) Quantification of DCX^+^ cells in the hilus of the DG of each group (*n* = 10 sections/5 mice/group). (**D**) Quantification of Prox1^+^ cells in the hilus of the DG of each group (*n* = 10 sections/5 mice/group). (**E**) Quantification of the density of DCX^+^ immunoreactivity in the DG of each group (*n* = 10 sections/5 mice/group). (**F**) Representative bands of DCX, Prox1, and α-tubulin proteins in the hippocampus. (**G**) Quantification of the ratio of DCX/α-tubulin and Prox1/α-tubulin by densitometric analysis. Data are expressed as the mean ± SEM (*n* = 5 mice/group). (**H**) Representative confocal images of the colocalization of DCX (red) with NeuN (green, a marker for mature neurons) in the DG of the hippocampus. Arrows indicate DCX^+^ cells in the hilus. Scale bar: 50 μm. (**I**) Categorization of dendritic morphology from DCX^+^/NeuN^+^ cells in the DG of the hippocampus. Category A, No processes; Category B, Short process; Category C, Medium process; Category D, Process reaching molecular layer; Category E, One dendrite branching in the molecular layer; Category F, Delicate dendritic tree branching in the GCL with quantification of the morphological distribution and number of total DCX^+^ cells. (**J**) Quantification of the number of DCX^+^ cells in each category. Data are expressed as the mean ± SEM (*n* = 5 mice/group). * *p* < 0.05, ** *p* < 0.01, **** *p* < 0.0001. CON, control mice; DAPI, 4′,6-diamidino-2-phenylindole; DCX, doublecortin; HFE, herbal formula extract; NeuN, neuronal nuclei; PCNA, proliferating cell nuclear antigen; Prox1, Prospero homeobox protein 1; PTSD, post-traumatic stress disorder; SEM, standard error of the mean.

The hippocampus is a brain region known to play an important role in learning and memory, affective behavior, mood regulation, and functional and structural neural plasticity [30]. Our previous paper demonstrated that PTSD-related neuroplastic remodeling, with structural and functional changes in the hippocampus, is associated with anxiety and memory impairment [28]. We aimed to investigate the structural changes and expression of proliferating cells and dentate granule cells (DGCs) in the hippocampus of PTSD mice and determine the effect of HFE. As shown in PCNA immunostaining, an indicator of cell proliferation in the subgranular zone (SGZ) of the DG, PCNA^+^ cells were significantly increased in the SGZ of PTSD mice compared with that in CON mice. However, HFE treatment reduced the PCNA^+^ cells (Figure 2A,B). We examined the expression pattern of DCX (a marker for immature DGCs) and Prox1 (a marker for mature DGCs) by immunostaining and Western blot to investigate changes in immature DGCs and mature DGCs in the DG. As shown in Figure 2A, some DCX^+^ cells and Prox1^+^ cells were located in the hilus of the DG in PTSD mice compared with those in CON mice (Figure 2A). The quantitative graph showed that the number of hilar DCX^+^ and Prox1^+^ cells was higher in PTSD mice than in CON mice (Figure 2C,D). As observed in immunostaining and Western blots, the expression of DCX^+^ cells in PTSD mice was less compared with that in CON mice, although it was restored by HFE treatment (Figure 2E,F).

Next, we examined the distribution of dendritic morphologies reflecting neuronal differentiation of DCX^+^ cells by category according to the study of Plümpe et al. [31]. In PTSD mice, most DCX^+^ cells were distributed in category CD (intermediate stage), with fewer DCX^+^ cells distributed in category EF (postmitotic stage) compared with those in CON mice (Figure 2H−J). However, HFE-treated mice showed significantly decreased DCX^+^ cells in category CD and increased DCX^+^ cells in category EF. These results indicate that treatment with HFE restored hippocampal DGC dysfunction, which was compromised in PTSD mice.

### 3.4. HFE Administration Reverses PTSD-Induced Reduction in the Reelin/Dab1 Pathway in the Hippocampus

**Figure 3:**
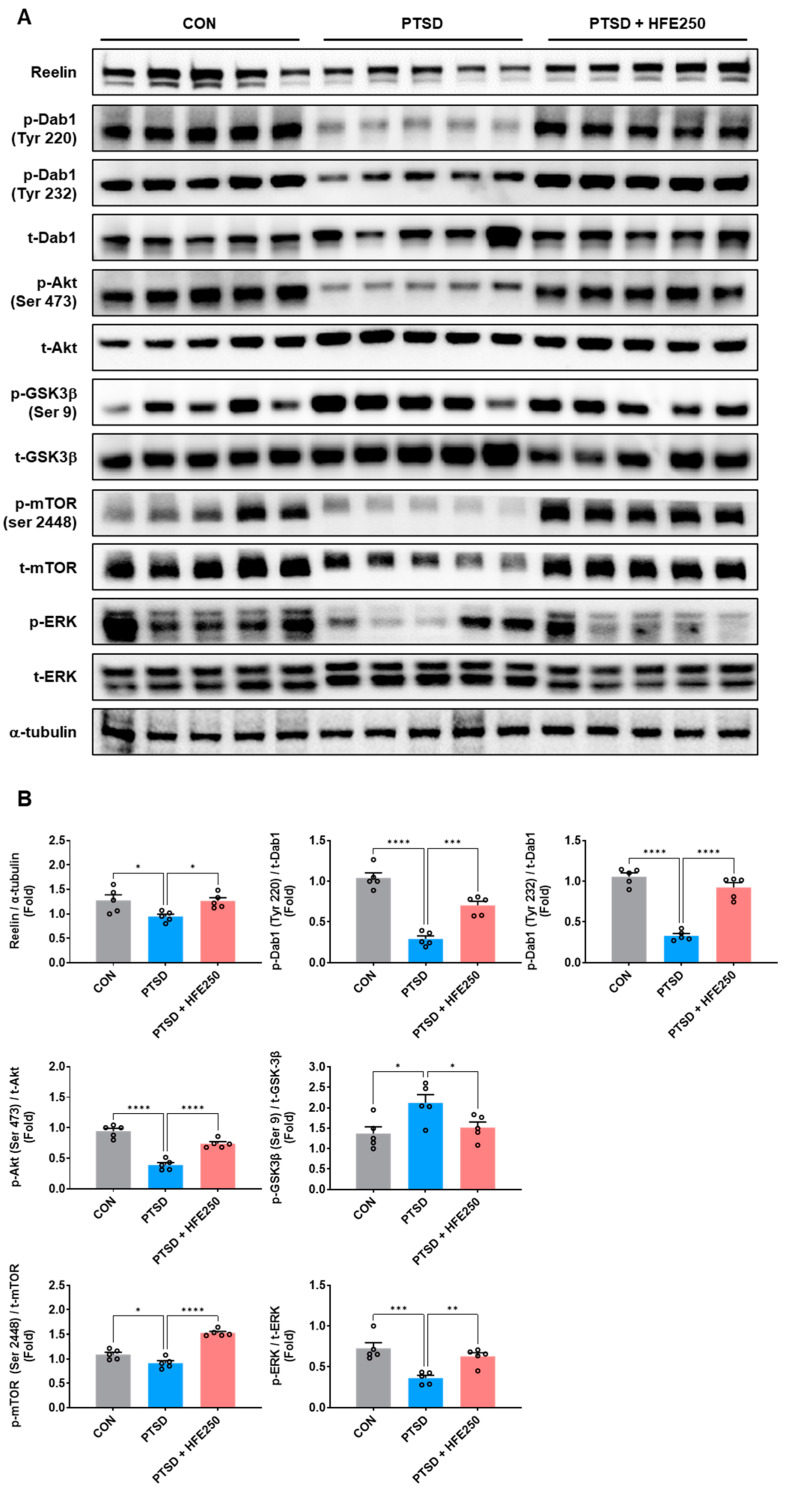

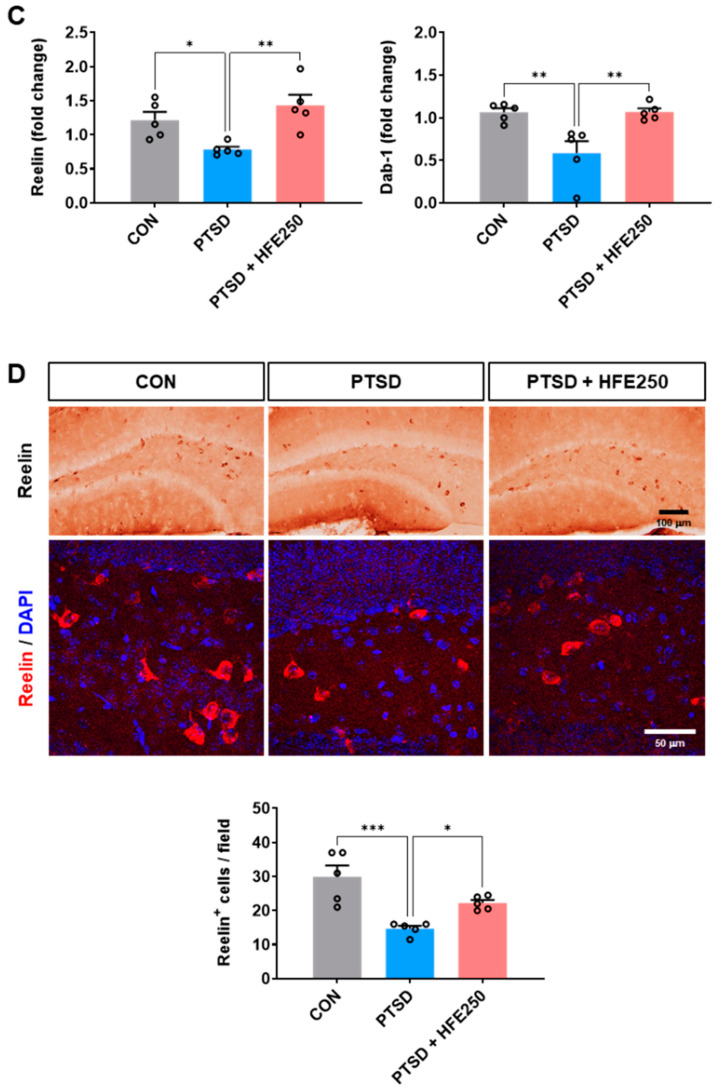
Administration of HFE increases the expression of the Reelin/Dab1 pathway in the hippocampus of PTSD mice. (**A**) Representative bands of Reelin/Dab1 pathway, related factors (p-Akt, t-Akt, p-GSK3β, t-GSK3β, p-mTOR, t-mTOR, p-ERK, and t-ERK), and α-tubulin proteins in the hippocampus. (**B**) Quantification of the ratio of Reelin/α-tubulin, p-Dab1/t-Dab1, p-Akt/t-Akt, p-GSK3β/t-GSK3β, p-mTOR/t-mTOR, and p-ERK/t-ERK by densitometric analysis. (**C**) Quantification of mRNA expression of *Reelin* and *Dab1* in the hippocampus. (**D**) Representative images of Reelin in the DG of the hippocampus. Scale bar: 100 μm or 50 μm. Quantification of Reelin^+^ cells in the DG. Data are presented as the mean ± SEM (*n* = 5 mice/group); * *p* < 0.05, ** *p* < 0.01, *** *p* < 0.001, **** *p* < 0.0001. Akt, protein kinase B; CON, control mice; Dab1, disabled-1; DAPI, 4′,6-diamidino-2-phenylindole; ERK, extracellular signal-regulated kinase; GSK3β, Glycogen synthase kinase-3β; HFE, herbal formula extract; mTOR, mammalian target of rapamycin; PTSD, post-traumatic stress disorder; SEM, standard error of the mean.

Reelin with Dab1, downstream signal transduction, is required for the proper positioning of neurons in the brain, modulates synaptic plasticity, and controls mood and cognitive function [11,32,33]. We examined the expression of Reelin and Dab1 pathways in the hippocampus of each group to determine which cellular pathway affects hippocampal dysfunction: increased hilar DGCs and/or decreased dendritic morphology. We found that the expression of Reelin and p-Dab1, downstream of the Reelin pathway, in the hippocampus was significantly reduced in PTSD mice compared with that in CON mice (Figure 3A,B). Experimental data showed that other downstream factors of the Reelin/Dab1 pathway, including p-Akt, p-GSK3β, p-mTOR, and p-ERK, were decreased in the hippocampus of PTSD mice compared with that of CON mice. Treatment with HFE significantly recovered the expression of Reelin and p-Dab1, as well as phosphorylation of downstream factors in the hippocampus. The mRNA expressions of *Reelin* and *Dab1* were also reduced in the hippocampus of PTSD mice. However, hippocampal mRNA expressions were restored by HFE treatment (Figure 3C). As shown in immunostaining for Reelin^+^ cells, they were mainly observed in the SGZ and hilus of the DG, and the number of Reelin^+^ cells, which was decreased in PTSD mice, was found to be elevated by HFE treatment (Figure 3D).

### 3.5. Treatment with HFE Restored Reelin Methylation of the Hippocampus in PTSD Mice

**Figure 4:**
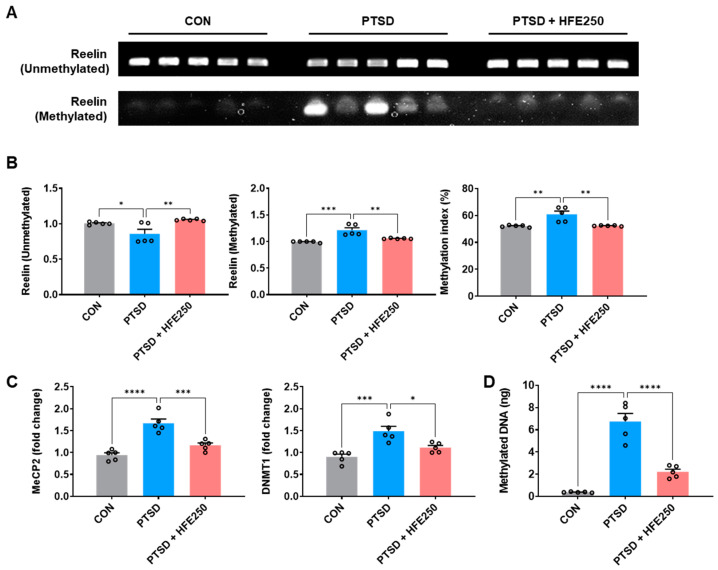
Treatment with HFE reduces the Reelin methylation and related factors in the hippocampus of PTSD mice. (**A**) Representative gel images were obtained from the hippocampus of each group using specific primers designed to detect unmethylated or methylated *Reelin*. (**B**) Quantification of band intensity of unmethylated or methylated *Reelin* and methylation index (%). (**C**) The quantification of mRNA expression of *MeCP2* and *DNMT1* in the hippocampus of each group. (**D**) The quantification of total DNA methylation in the hippocampus of each group. Data are presented as the mean ± SEM (*n* = 5 mice/group); * *p* < 0.05, ** *p* < 0.01, *** *p* < 0.001, **** *p* < 0.0001. CON, control mice; DNMT1, DNA methyltransferase 1; HFE, herbal formula extract; MeCP2, methyl-CpG binding protein 2; PTSD, post-traumatic stress disorder; SEM, standard error of the mean.

We examined DNA methylation of the *Reelin* gene using the bisulfite modification method to investigate whether it is epigenetically regulated in the hippocampus of PTSD mice. We observed relatively higher methylation of *Reelin* in PTSD mice than in CON mice and decreased methylation in HFE-treated mice (Figure 4A,B). The increase in *Reelin* methylation index in the hippocampus of PTSD mice ranged from 6.8 to 10.8% compared with that in CON mice (*p* < 0.01), and it was reduced in HFE-treated mice (Figure 4A,B). We examined the factors involved in DNA methylation, such as MeCP2 and DNMT1 methyl-CpG binding protein 2 and DNA methyltransferase 1, in the hippocampus of each group to determine whether the methylation of the *Reelin* involves changes in the methylation-related factors. RT-qPCR analysis of MeCP2 and DNMT1 showed that mRNA levels of *MeCP2* and *DNMT1* were significantly increased in the hippocampus of PTSD mice (Figure 4C). Treatment with HFE decreased mRNA expression of *MeCP2* and *DNMT1* in the hippocampus (Figure 4C). Because PTSD induced increases in MeCP2 and DNMT1 expression, we next determined DNA methylation in the hippocampus of each group using a global DNA methylation assay. Methylated DNA significantly increased in the hippocampus of PTSD mice compared with that in CON mice; however, it decreased after HFE treatment (Figure 4D).

### 3.6. Treatment with HFE Increases the Expression of Synaptic Plasticity-Associated Genes in the Hippocampus of PTSD Mice

**Figure 5:**
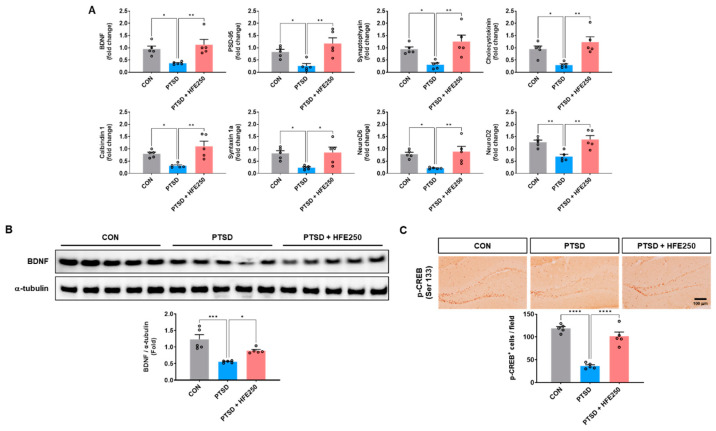
Treatment with HFE increases the expression of synaptic plasticity-related factors in the hippocampus of PTSD mice. (**A**) The quantification of mRNA expression of hippocampal function-related genes in the hippocampus. (**B**) Representative bands of BDNF and α-tubulin proteins in the hippocampus. Quantification of the ratio of BDNF/α-tubulin by densitometric analysis. (**C**) Representative images of p-CREB in the DG of the hippocampus. Scale bar: 100 μm. Quantification of p-CREB^+^ cells in the DG. Data are presented as the mean ± SEM (*n* = 5 mice/group); * *p* < 0.05, ** *p* < 0.01, *** *p* < 0.001, **** *p* < 0.0001. BDNF, brain-derived neurotrophic factor; CON, control mice; CREB, cyclin AMP-response element-binding protein; HFE, herbal formula extract; PTSD, post-traumatic stress disorder; SEM, standard error of the mean.

We determined whether there were any changes in gene expression associated with synaptic plasticity due to a PTSD-induced increase in DNA methylation in the hippocampus using RT-qPCR to assess gene expression profiles associated with neural plasticity and memory function. This analysis included brain-derived neurotrophic factor (BDNF), postsynaptic density protein-95 (PSD-95), synaptophysin, cholecystokinin, calbindin 1, syntaxin 1a, neuronal differentiation 6 (NeuroD6), and neuronal differentiation 2 (NeuroD2) (Figure 5A). The mRNA levels of synaptic plasticity-related genes were reduced in the hippocampus of PTSD mice but increased by HFE treatment. (Figure 5A). Western blot analysis and immunostaining also showed decreases in the protein expression of BDNF and p-CREB in the hippocampus of PTSD mice (Figure 5B,C). We found that mice treated with HFE showed higher BDNF and p-CREB expression than did PTSD mice (Figure 5B,C).

### 3.7. Identification of Active Compounds of HFE

**Figure 6:**
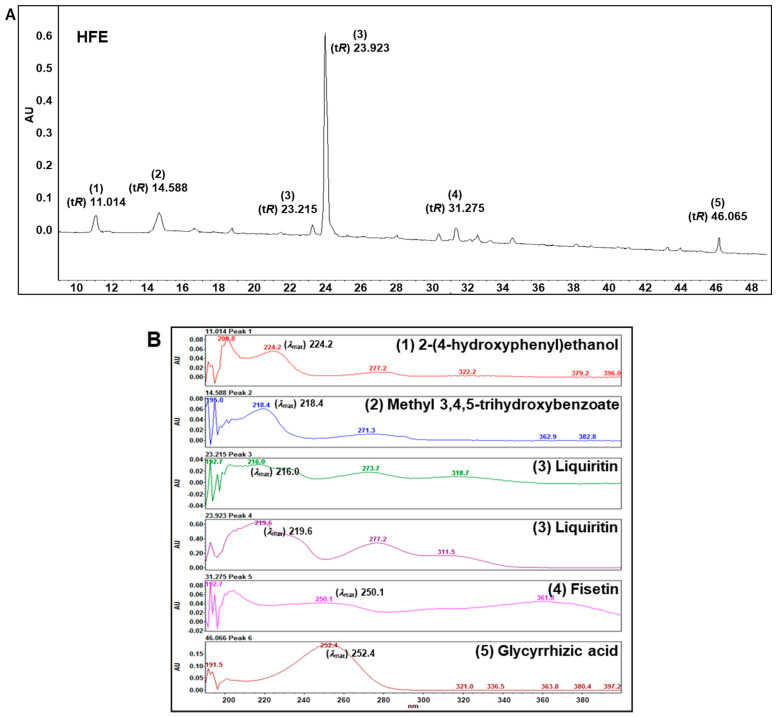

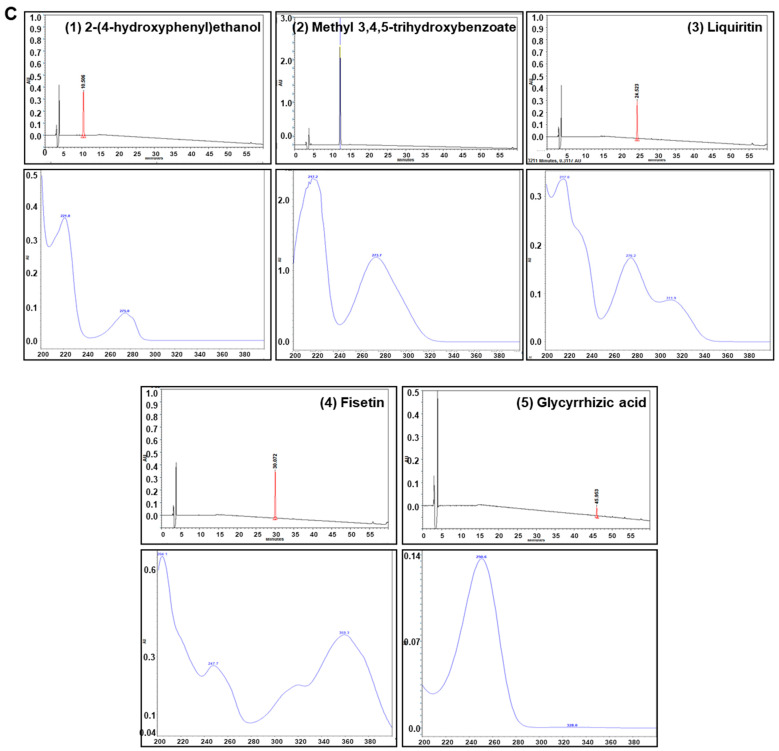
Active compounds in HFE. Representative HPLC/PDA chromatograms showing the retention time of each peak in HFE (**A**), λmax of each peak in HFE (**B**), and active compounds (**C**). Peaks (1) 2-(4-hydroxyphenyl)ethanol, (2) methyl 3,4,5-trihydroxybenzoate, (3) liquiritin, (4) fisetin, and (5) glycyrrhizic acid from HFE. HPLC/PDA, high performance liquid chromatography/photodiode array detector; HFE, herbal formula extract; t*R*, retention time; λmax, UV absorption maxima; SEM, standard error of the mean.

Qualitative analysis of active compounds of HFE was performed using HPLC/PDA chromatograms. The active compounds in HFE were identified by comparison of retention time (t*R*) and UV absorption maxima (*λ_max_*) with commercial standards (Figure 6). Based on these results, peaks 1−5 were allocated as 2-(4-hydroxyphenyl)ethanol (1), methyl 3,4,5-trihydroxybenzoate (2), liquiritin (3), fisetin (4), and glycyrrhizic acid (5).

## 4. Discussion

In this study, we found that PTSD mice exhibited behavioral impairment as well as structurally and morphologically abnormal DGCs, including increased hilar ectopic DGCs and decreased dendritic morphologies for neuronal differentiation in the hippocampus. In addition, HFE effectively blocked PTSD-induced behavioral deficits and hippocampal dysfunction. The cellular mechanisms associated with the abnormal neuronal responses and behavior deficits seen in the hippocampus of PTSD are clearly identified; moreover, treatments that can effectively block them have not been explored sufficiently. In this study, we showed the anxiolytic and cognition-enhancing effects of HFE on PTSD-induced hippocampal dysfunction, via upregulation of the Reelin/Dab-1 pathway, as well as reduction in DNA methylation in the hippocampus. To the best of our knowledge, this study is the first to investigate the specific effect of a novel HFE in mice with PTSD.

PTSD is a stress-related mental disorder generally occurring in response to complex traumas, including prolonged or repetitive exposures to traumatic events [34]. Currently, prescribed medications for PTSD are limited in their efficacy to cover complex symptoms because of the complex PTSD phenotypes. Accordingly, it is crucial to explore herbal treatments that can alleviate PTSD symptoms because they offer a range of mechanisms and active compounds for this purpose. In this study, we designed a novel combination of herbal medicines, including *P. ginseng*, *A. membranaceus*, *A. macrocephala*, and *G. uralensis*, and revealed the efficacy of HFE in PTSD mice. These three herbal species are well-recognized for their neuroprotective effects via the regulation of inflammation, oxidative stress, and neurogenic mechanisms [23,24,25,26]. In addition, they contain a variety of bioactive compounds, including ginsenoside Rb1, astragaloside, atractylenolide, and licorice, which have shown efficacy against anxiety, depression, and PTSD [35,36,37,38].

The hippocampus is located within the medial part of the temporal lobe and plays a major role in memory, learning, anxiety, and emotion. Hippocampal dysfunction, including plasticity, cellular structure, and epigenetic changes, may be sensitive to stress responses and ultimately cause memory decline and anxiety disorders [39]. In this study, we used a modified PTSD mouse model created by SPS and FS. The serum of PTSD mice showed increased levels of corticosterone and decreased levels of serotonin; this suggested that the PTSD mouse model was well-established. We observed that PTSD mice tended to prefer the non-center zone over the central zone in the OFT, with reduced spontaneous alternation in the Y-maze test. As shown in the OFT and Y-maze test, the total distance traveled and number of entries decreased, indicating a decrease in locomotor activity due to PTSD-induced anxiety. Higher spontaneous alternation rates in mice indicate improved cognitive performance, as they effectively remember and choose new arms. Thus, the Y-maze test can provide critical insights into how PTSD affects cognitive function. Therefore, reduced spontaneous alternations in the Y-maze test suggest deficits in cognitive function in mice, likely caused by structural abnormalities in the hippocampus, such as those observed in DGCs. Our results indicated that PTSD mice showed anxiety-like behavior and cognitive impairment. However, HFE treatment significantly alleviated these behavioral abnormalities. Behavior abnormalities such as anxiety and memory loss are well-known key features of PTSD in humans and the rodent model [40,41]. Therefore, our findings suggest that HFE could be an effective anxiolytic and memory-improving treatment for PTSD.

Neuroplasticity is represented by changes at structural and functional levels and is characterized by the expression of plasticity-related factors, which affect brain development and homeostasis, memory formation, and neurocircuitry [42]. Structural changes are related to the changes in dendritic arborization, density of dendritic spines, and synaptic structure of neurons. Functional plasticity indicates LTP and long-term depression (LTD). Several studies have reported that impaired neuroplasticity may be one of the pathological mechanisms for PTSD [43,44]. Hippocampal LTP as well as plasticity-related factors were significantly reduced in animal models of PTSD [45,46]. Our previous study showed that PTSD mice exhibited hyperactivation and aberrant localization of DGCs compared with CON mice [28]. This study allowed us to identify additional evidence related to neuroplasticity impairments in PTSD mice and to demonstrate the effectiveness of the HFE. The proliferating cells (PCNA^+^ cells) and hilar ectopic migration (hilar Prox1^+^ cells and hilar DCX^+^ cells) in the DG were found to be localized in the hippocampus of the PTSD mice in this study. In addition, suppressed morphological maturation from analysis of the distribution of dendritic morphologies among DCX^+^ cells in the DG was observed in PTSD mice, reflecting reduced protein expression of DCX in the hippocampus. However, HFE treatment restored the impairment of hippocampal neuroplasticity in PTSD mice. Hilar ectopic DGCs are hyperexcitable and contribute to increased circuit excitability in environmental stress or brain injury [4,47]. Therefore, our results demonstrate that this HFE could be an effective neuroplasticity modulator in PTSD.

Reelin, an extracellular matrix protein, is involved in the cellular mechanisms that regulate hippocampal function and has important functions in the brain, including the modulation of neuronal migration, dendritic growth, dendritic spine formation, and synaptic plasticity, resulting in the regulation of memory and anxiety [48]. Many brain disorders, such as schizophrenia, temporal lobe epilepsy, depression, autism, and neurodegenerative disease, have been associated with dysregulated Reelin expression [49]. Genetic deletion- or chronic stress exposure-induced Reelin reduction in the hippocampus has been reported to exhibit suppressed synaptic plasticity and increased depression-like behavior and cognitive deficits [15,16,17,18]. In addition, Dab-1 conditional knockout mice exhibited hyperactive behaviors and impairment of working memory, which are symptoms observed in patients with psychiatric disorders [50]. Therefore, Reelin and its pathways serve as key cellular mechanisms for understanding hippocampal dysfunction and behavioral abnormalities caused by PTSD and as putative targets for developing treatments. Our results from Western blot analysis, RT-qPCR, and immunostaining showed that Reelin, Dab-1, and downstream proteins (Akt, GSK3β, mTOR, and ERK) are regulated by HFE treatment, which significantly decreased in the hippocampus of PTSD mice. Previous studies reported that mice with chronic stress induced by corticosterone injection or social stress exhibited a reduction in Reelin and synaptic plasticity-related protein expression in the hippocampus [51,52,53]. Interestingly, Brymer et al. [54] suggested that exogenous Reelin injection into the dorsal hippocampus in repeated corticosterone-injected stressor mice can restore memory deficits and hippocampal dysfunction.

DNA methylation is an epigenetic mechanism that modulates gene transcription and is involved in memory formation and maintenance by the regulation of genomic responses in hippocampal neurons, particularly in neuroplasticity and LTP [55,56]. Epigenetic modifiers such as MeCP2 and DNMT1 mediate DNA methylation [57]. Methylated CpG sites within gene promoters are considered markers for the prediction of various diseases, including psychiatric disorders [58,59]. Given the epigenetic factors in PTSD, several studies have focused on the role of DNA methylation in PTSD-related symptoms. Peripheral blood samples of patients with PTSD showed higher DNA methylation than did those of patients without PTSD, indicating that DNA methylation is highly correlated with PTSD-related symptoms, including the decline in cognitive function [60,61]. Building on this evidence, our study aimed to investigate the effects of HFE on methylated DNA levels and epigenetic modifiers in PTSD mice. Our findings revealed that the methylation index of *Reelin* was significantly increased in the hippocampus of PTSD mice compared to that in CON mice. In addition, mRNA levels of *MeCP2* and *DNMT1* were elevated, and there was a global hypermethylation of DNA in the hippocampus in the PTSD mice compared to those in CON mice, consistent with previous findings in patients with PTSD [62,63]. Mice with HFE treatment showed a significant decrease in *Reelin* methylation, mRNA levels of *MeCP2* and *DNMT1*, and global DNA methylation in the hippocampus. The demethylation of promoters results in gene activation, increased transcriptional activity, and protein synthesis [64]. In this study, we investigated whether the Reelin/Dab-1 pathway also regulates the expression of synaptic plasticity-related genes. Hippocampal function-related genes (*BDNF*, *PSD-95*, *Synaptophysin*, *Cholecystokinin*, *Calbindin 1*, *Syntaxin 1a*, *NeuroD6*, and *NeuroD2*) were significantly downregulated in the hippocampus of PTSD mice, although they were restored by HFE treatment. Protein expression of BDNF and pCREB in the hippocampus, which are also associated with hippocampal function, was regulated by HFE treatment in PTSD mice. Pharmacological inhibition of changes in DNA methylation affects synaptic plasticity, learning, and memory. Previous studies reported that inhibition of DNA methylation by injection of DNMT (5-aza-2-deoxycytidine; 5-AZA) ameliorated spatial learning and memory in hypoxic mice or mice with chronic unpredictable mild stress through restored BDNF and BDNF pathway expression [65,66]. In addition, Chang et al. [67] reported that 5-AZA-treated mice improved conditioned avoidance learning and hippocampal neurogenesis by the Notch1 pathway. Therefore, our results suggest that HFE acts as an inhibitor of DNA methylation, especially *Reelin* methylation, to restore hippocampal function, anxiety, and cognitive function by increasing hippocampal function-related gene transcription.

We found five active compounds in HFE: 2-(4-hydroxyphenyl)ethanol, methyl 3,4,5-trihydroxybenzoate, liquiritin, fisetin, and glycyrrhizic acid. These compounds have been reported to be effective as therapeutic agents for depression, anxiety, and memory loss by providing protection against oxidative stress and neuroinflammation. Methyl 3,4,5-trihydroxybenzoate and 2-(4-hydroxyphenyl)ethanol have anti-oxidant activity and anti-amyloidogenic activity by attenuating Amyloid β accumulation in Alzheimer’s disease mouse model and Neuro2A cells [68,69]. Ma et al. [70] reported that glycyrrhizic acid treatment reduced chronic restraint stress-induced anxiety by restoring glutamate re-uptake and circadian rhythm gene expression. Liquiritin and fisetin also exert anti-depressive effects and anxiolytic effects by regulating oxidative stress and neuroinflammation in the brain [71,72].

## 5. Conclusions

In conclusion, we demonstrated that HFE could help ameliorate anxiety-like behavior and cognitive deficit by restoration of hippocampal dysfunction, including plasticity and cellular and epigenetic changes, via regulation of the Reelin pathway and DNA methylation. Our study suggests that the Reelin pathway and DNA methylation are associated with the therapeutic effects of HFE in the hippocampus of PTSD mice. Thus, our study demonstrates that HFE could be regarded as a potential therapeutic agent for PTSD and other psychological disorders. Nonetheless, additional studies are needed to examine other epigenetic modulations, including DNA methylation of other genes, as well as histone modifications and chromatin remodeling in PTSD. Further investigations are needed to evaluate the therapeutic effects of HFE in clinical studies involving patients with PTSD. In addition, approaches are needed to explore other brain regions involved in PTSD symptoms.

## Figures and Tables

**Table 1:** Primer sequences used for RT-qPCR and bisulfite conversion-based PCR.

Name	Primer and Sequence (5′-3′)
*Reelin*	Forward-TCACTGTGTCATACGCCAAGAAC Reverse-GAGGTACAGGATGTGGATGACTGT
*Dab-1*	Forward-CTAGGCAGAGCTCTCCATCC Reverse-GACTTATATTATCACCACTGGGCTC
*MeCP2*	Forward-CAGCAGCATCTGCAAAGAAG Reverse-TCCACAGGCTCCTCTCTGTT
*DNMT1*	Forward-GAGTCTTCGACGTCACACCA Reverse-AGCTACCTGCTCTGGCTCTG
*BDNF*	Forward-TTACTCTCCTGGGTTCCTGA Reverse-ACGTCCACTTCTGTTTCCTT
*PSD-95*	Forward-GCTCCCTGGAGAATGTGCTA Reverse-TGAGAAGCACTCCGTGAACT
*Synaptophysin*	Forward-GCCTACCTTCTCCACCCTTT Reverse-GCACTACCAACGTCACAGAC
*Cholecystokinin*	Forward-ATACATCCAGCAGGTCCGCAA Reverse-CAGACATTAGAGGCGAGGGGT
*Calbindin 1*	Forward-TCTGGCTTCATTTCGACGCTG Reverse-ACAAAGGATTTCATTTCCGGTGA
*Syntaxin 1a*	Forward-CCGAACCCCGATGAGAAGAC Reverse-TGCTCTTTAGCTTGGAGCGA
*NeuroD6*	Forward-ATGCGACACTCAGCCTGAAA Reverse-CTGGGATTCGGGCATTACGA
*NeuroD2*	Forward-AAGCCAGTGTCTCTTCGTGG Reverse-TTGGACAGCTTCTGCGTCTT
*GAPDH*	Forward-CCTCGTCCCGTAGACAAA Reverse-AATGAAGGGGTCGTTGATG
*Reelin (unmethylated)*	Forward-TTTTTAGTAATGTGTAAATATAGAGTTTGG Reverse-AAATAATACAAAACCAAATCATCAAA
*Reelin (methylated)*	Forward-TTAGTAACGCGTAAATATAGAGTTCG Reverse-ATAATACGAAACCAAATCGTCGA

Abbreviations: BDNF, brain derived-neurotrophic factor; Dab-1, disabled-1; DNMT1, DNA methyltransferase 1; GAPDH, Glyceraldehyde 3-phosphate dehydrogenase; MeCP2, methyl CpG binding protein 2; NeuroD2, neuronal differentiation 2; NeuroD6, neuronal differentiation 6; PSD-95, postsynaptic density protein-95.

## Data Availability

The datasets supporting the conclusions in this study are included in the article.
